# Cardiopulmonary Support During Catheter Ablation of Ventricular Arrhythmias: Long-Term Results from a Single-Center Experience

**DOI:** 10.3390/jcdd13080365

**Published:** 2026-08-03

**Authors:** Davide Ciliberti, Antonio Di Monaco, Federico Quadrini, Federica Troisi, Nicola Vitulano, Luca Sgarra, Elia Iorio, Marcello Martimucci, Nicola Caporusso, Giovanna Magnesa, Fabrizia Massaro, Rosa Caruso, Nicola Duni, Vincenzo Anzelmo, Alberto Martinelli, Francesco Mangini, Salvatore Maurizio Maggiore, Paola Pierucci, Massimo Grimaldi

**Affiliations:** 1 Department of Medicine and Surgery, LUM University, Casamassima, 70010 Bari, Italy; s.maggiore@miulli.it (S.M.M.); p.pierucci@miulli.it (P.P.); m.grimaldi@miulli.it (M.G.); 2 Department of Cardiology, Miulli General Regional Hospital, Acquaviva Delle Fonti, 70021 Bari, Italy; f.quadrini@miulli.it (F.Q.); f.troisi@miulli.it (F.T.); n.vitulano@miulli.it (N.V.); l.sgarra@miulli.it (L.S.); e.iorio@miulli.it (E.I.); m.martimucci@miulli.it (M.M.); rcaruso25@yahoo.it (R.C.); n.duni@miulli.it (N.D.); v.anzelmo@miulli.it (V.A.); albertomartinelli4@icloud.com (A.M.); f.mangini@miulli.it (F.M.); 3 Department of Anesthesia and Intensive Care, Miulli General Regional Hospital, Acquaviva Delle Fonti, 70021 Bari, Italy; n.caporusso@miulli.it (N.C.); g.magnesa@miulli.it (G.M.); f.massaro@miulli.it (F.M.); 4 Department of Pneumology, Miulli General Regional Hospital, Acquaviva Delle Fonti, 70021 Bari, Italy

**Keywords:** ventricular arrhythmias, catheter ablation, extracorporeal membrane oxygenation

## Abstract

Extracorporeal membranes oxygenation (ECMO) has been proposed as a useful tool to support ablation of unstable ventricular arrhythmias (VAs). The aim of this study is to assess the clinical outcome of cardiopulmonary support of VAs catheter ablation during a long-term follow-up. In this retrospective observational study, we included 47 patients referred to our center for catheter ablation of repeated episodes of hemodynamically unstable sustained VAs between April 2016 and February 2025. All patients underwent catheter ablation, supported by ECMO, of ventricular arrhythmias symptomatic for syncope or presyncope. The primary endpoint is overall cardiovascular death, including death due to heart failure, cardiogenic shock or ventricular arrhythmias. In particular, arrhythmic death was defined as death occurring during an electrical storm. After a median follow-up of 28 (7–63.5) months, cardiovascular death occurred in 26 patients (55.3%) but arrhythmic death befell only 11 patients (23.4%). All deaths occurred within 6–7 years of follow-up. No difference exists between ischemic and non-ischemic cardiomyopathy with regard to primary endpoints. Moreover, arrhythmic recurrences occurred in 21 patients (44.7%), among whom only 15 (31.9%) had ICD shocks; 25 patients (55.3%) encountered further hospitalizations. ECMO may facilitate procedural mapping and acute ablation success in selected high-risk patients, while long-term prognosis remains mainly driven by advanced heart failure.

## 1. Introduction

Ventricular arrhythmias (VAs) are often life-threatening [1] and the risk of death and poor outcome is even higher when they are clustered or organized as an electrical storm (ES) [2,3,4,5].

VAs could occur in structural diseases and primary electrical diseases. In the first case they could represent a worsening phase of the disease and the underlying heart failure, especially in the presence of complex cardiac substrate, reduced left ventricular mechanical function and comorbidities, whereas in primary electrical diseases, ventricular arrhythmias are elicited by ion channel disorders and could be considered as the main signs of the disease, in the absence of a structural substrate [1].

According to the latest guidelines, antiarrhythmic drugs are the first-line therapy, followed by catheter ablation [1]. Indeed, catheter ablation can reduce recurrent episodes of ventricular arrhythmias and improve patient prognosis [3,6,7,8,9,10,11,12]. But ventricular arrhythmias may determine hemodynamic impairment, cardiogenic shock and cardiac arrest by themselves or in adjunction with the underlying mechanical failure and clinical conditions [13,14]. Thus, some of these patients, above all, those with unstable VAs and ES, are at higher risk of procedural complications and death, due to repetitive arrhythmias and multiple implantable cardioverter defibrillator intervention.

Recent data suggest that cardiopulmonary support with extracorporeal membrane oxygenation (ECMO) can provide valuable support for these patients, particularly during catheter ablation procedures [15,16,17,18,19,20,21]. Indeed, the ECMO system is useful for managing intraoperative acute hemodynamic decompensation and can facilitate the accurate mapping and ablation of ventricular arrhythmias [20,21,22]. In the literature, data concerning long-term outcome of patients who underwent VAs catheter ablation supported by ECMO are lacking. The aim of this study is to assess outcomes and report our experience using cardiopulmonary support to perform ventricular arrhythmias ablation in high-risk patients.

## 2. Materials and Methods

### 2.1. Study Population

In this retrospective observational study, we included patients referred to our center, Miulli General Regional Hospital, for catheter ablation of repeated episodes of hemodynamically unstable sustained VAs between April 2016 and February 2025. In particular, we included all the patients that underwent catheter ablation supported by ECMO. The PAINESD score, useful to evaluate the risk of acute hemodynamic decompensation during catheter ablation, was calculated for all patients [13]. ES was defined as the occurrence of three or more ventricular tachycardia/ventricular fibrillation episodes requiring electrical cardioversion or defibrillation in a 24 h period. All arrhythmias were unresponsive to amiodarone or other antiarrhythmic drugs.

The study was approved by the local Ethics Committee and complies with the Declaration of Helsinki. All participants provided written informed consent.

### 2.2. Electrophysiological Study and Catheter Ablation Supported by ECMO

**Figure 1:**
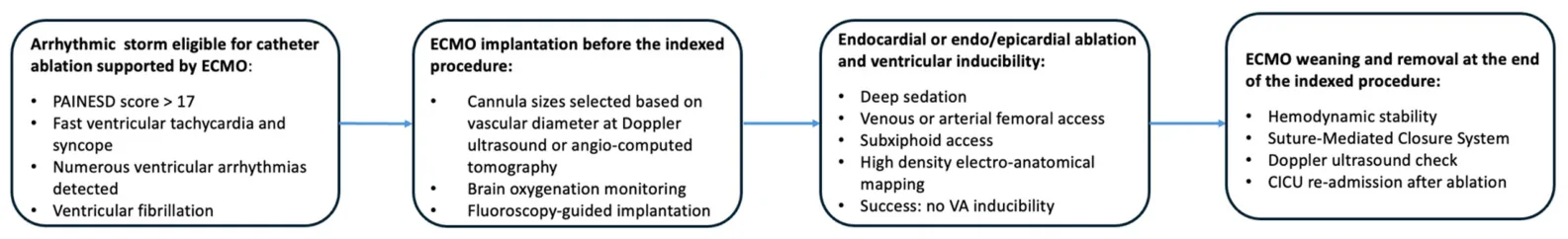
Procedural workflow of ventricular arrhythmia ablation supported by extracorporeal membrane oxygenation. VAs: ventricular arrhythmias. CICU: cardiac intensive care unit.

All procedures were performed by expert operators; the surgical team consisted of two electrophysiologists, one interventional cardiologist, one anesthesiologist, two perfusion technicians, and two nurses. Two vascular surgeons were also present if a patient required femoral artery isolation. The procedural workflow is summarized in Figure 1.

All patients underwent preoperative Doppler ultrasound and/or angio-computed tomography of the lower leg to assess the femoral arteries.

All procedures were initiated under conscious sedation with an intravenous infusion of dexmedetomidine 1 µg/Kg/h and fentanyl (0.2 mg) under the supervision of an anesthesiologist; general anesthesia was administered at the discretion of the anesthesiologist. Antibiotic prophylaxis (cefazolin 2 g and teicoplanin 400–600 mg) was administered immediately before the procedure. Intra-arterial blood pressure monitoring and digital pulse oximetry were monitored continuously during the procedure; ICD therapies were inactivated for the duration of the procedure. Patients were monitored using the Masimo Sedline (Masimo Corporation, Irvine, CA, USA), determining level of sedation or anesthesia.

**Figure 2:**
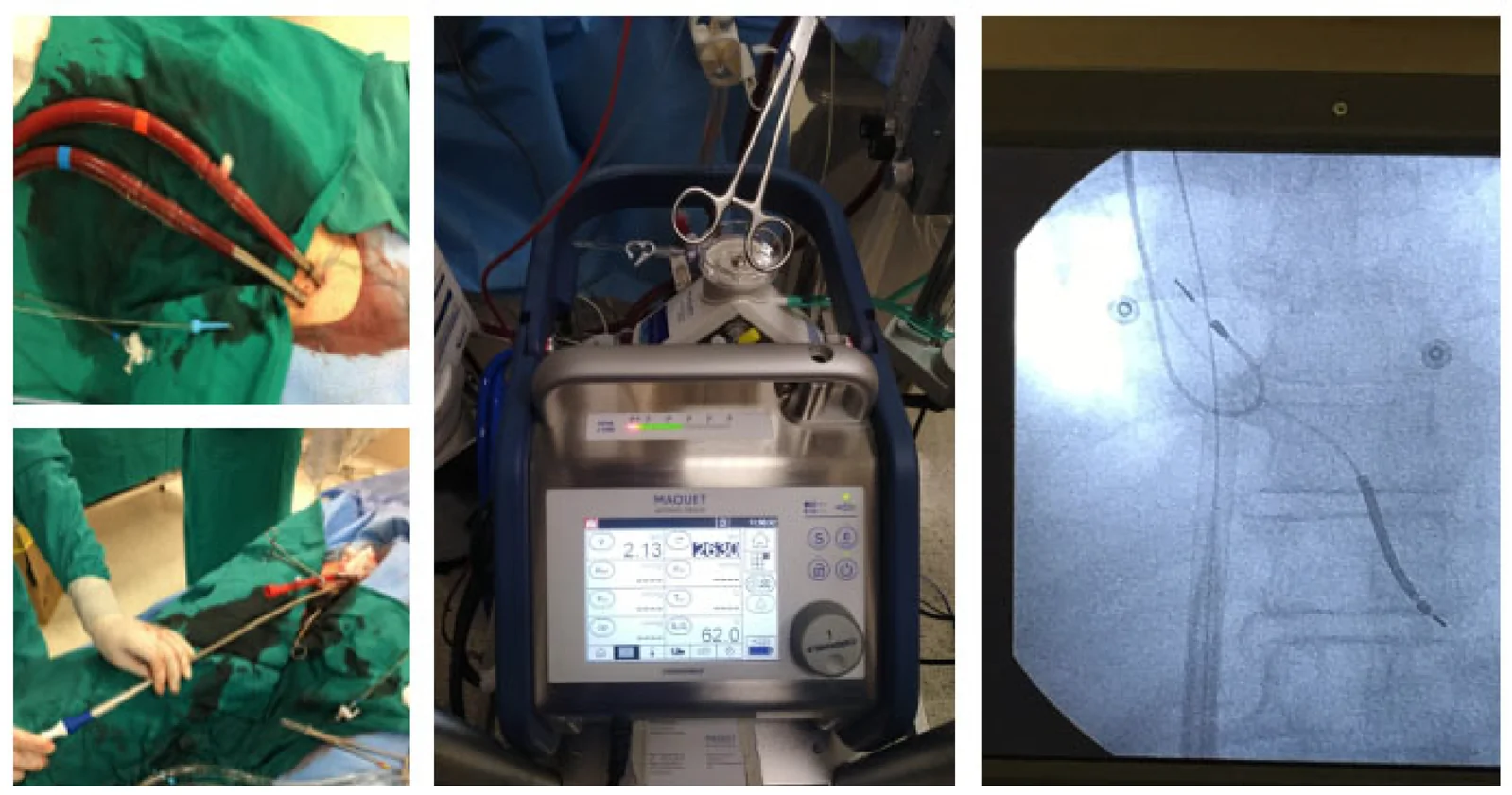
ECMO implantation (**right**), control panel (**center**), and angiographic view of venous cannula (**left**).

Before inserting ablation catheters into the heart, cannulas for ECMO support were positioned under the supervision of two expert perfusionists (Figure 2). The circuit (Cardiohelp System, Maquet, Rastatt, Germany) consisted of a centrifugal pump, polymethylpentene gas exchanger, heat exchanger, tubing, and variously sized cannulas for venous and arterial cannulation. The appropriate cannula sizes were selected based on an evaluation of vascular diameter from Doppler ultrasound or angio-computed tomography of the lower leg and on patient weight. The left femoral artery was cannulated and a guidewire was positioned in the right femoral artery. Angiography was performed to visualize the right common femoral artery and the artery was cannulated under fluoroscopic guidance using the Seldinger technique. Two Perclose Proglide (Abbot, North Chicago, IL, USA) suture-mediated closure systems were positioned in the femoral arteries to facilitate closure of the arteries at the end of the procedure. The right femoral vein was also cannulated. The arterial cannula was inserted into the common femoral artery and advanced up to the iliac artery. The venous cannula was advanced up to the right atrium under fluoroscopic guidance. In patients with a small right femoral artery, a small sheath was placed in the superficial femoral artery to permit distal flow and prevent limb ischemia. In four patients with significant femoral arterial atherosclerosis, the ECMO cannulas were positioned by vascular surgeons. ECMO support was started at 3 L and adjusted following the patient’s hemodynamics. During support, heparin was administered to a target-activated clotting time of 300 s in cases of endocardial left VAs or 250 s in cases of endocardial right or epicardial VAs.

In patients with VAs of suspected epicardial origin, a pericardial approach was guaranteed before positioning the ECMO circuit. This workflow was adopted so that the pericardial approach was performed before heparin administration. We performed the pericardial approach, as described previously [23], inserting a steerable sheath (Agilis, St. Jude Medical, Minnetonka, Minnesota, United States) in the pericardial space to allow catheter stability and maneuverability.

In all patients, the ablation catheter was inserted through the femoral artery/vein and located inside of the left/right ventricle or epicardium through the pericardial sheath. Mapping and ablation were performed with a 3.5 mm irrigated catheter with a contact force sensor (Thermocool Smartouch Surround Flow, Biosense Webster, Irvine, CA, USA) or a high-energy, temperature-controlled ablation catheter (QDOT Micro, Biosense Webster, Irvine, CA, USA) and a three-dimensional mapping system (CARTO, Biosense Webster, Inc., Irvine, CA, USA). Substrate maps were obtained using a multipolar mapping catheter (Pentaray, Octaray or Optrell; Biosense Webster, Irvine, CA, USA).

At first, a geometry of the chamber of interest was created using the ablation or mapping catheter; then, a substrate map was acquired during sinus rhythm or right ventricular pacing in pacing-dependent patients. In those patients with cardiac resynchronization therapy devices, left ventricular pacing was turned off.

**Figure 3:**
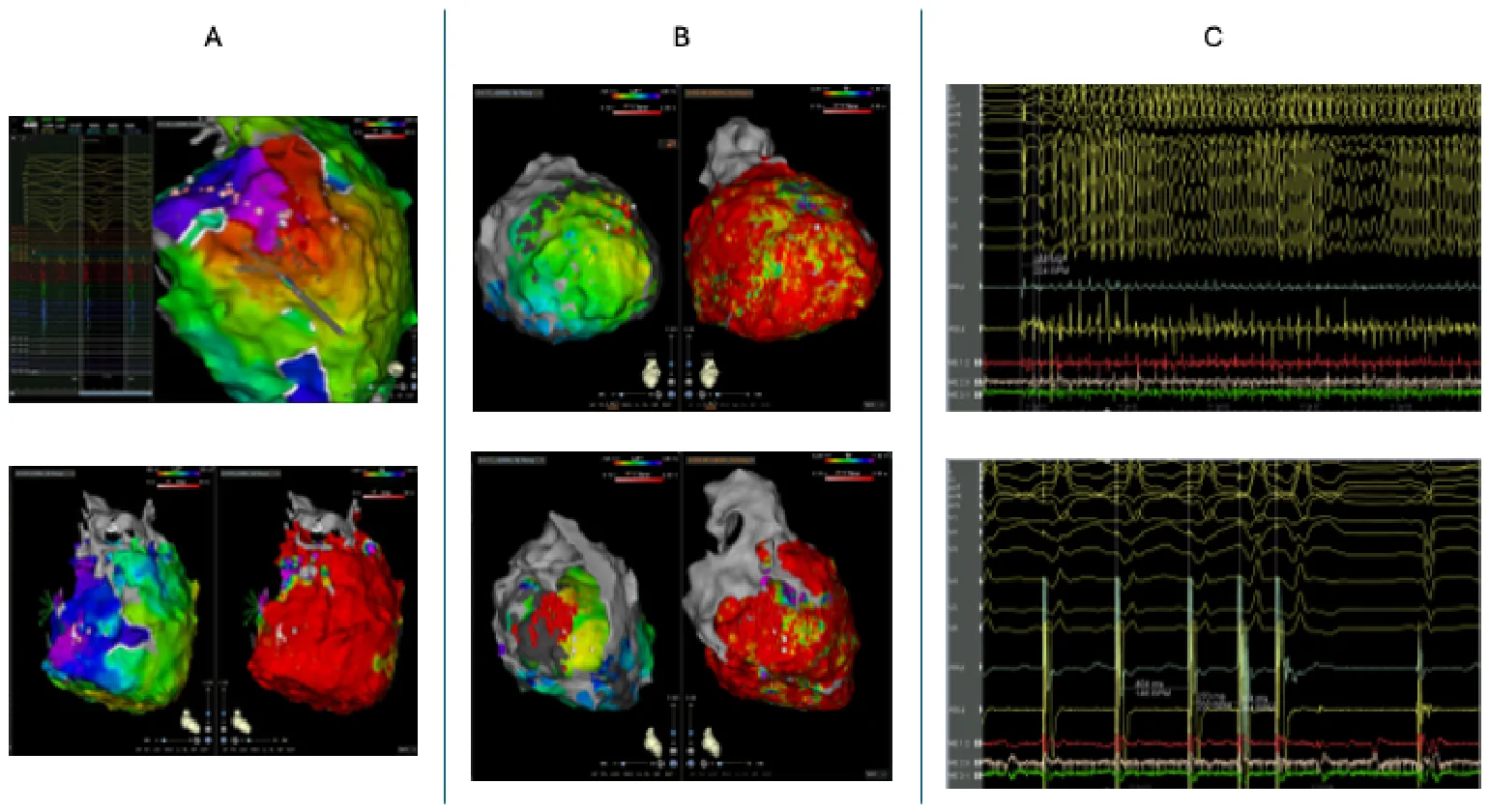
(**A**) Example of mapping and ablation of ventricular tachycardia in an inflammatory cardiomyopathy patient. (**B**) Example of mapping and ablation of ventricular tachycardia in a biventricular arrhythmogenic cardiomyopathy patient. (**C**) Ventricular fibrillation inducibility in the upper panel; no more ventricular arrhythmias were inducible after Purkinje premature ventricular contraction as shown in the lower panel.

Initially, an accurate substrate ablation was performed targeting the areas of local abnormal ventricular activity and late potentials [24,25,26] (Figure 3). In patients with a spontaneous induction of clinical VTs during substrate mapping or ablation, activation mapping was attempted if there was hemodynamic stability. Activation and entrainment mapping were performed to identify critical sites of the VT reentrant circuit as previously described [26,27,28,29]. In particular, the window of interest was opened from the termination of the first QRS to the onset of the second QRS of the VT cycle, to define the diastolic interval. The last step was ventricular inducibility. Programmed ventricular stimulation after ablation was performed at the right ventricular apex (basal drive 600/500/400 ms up to three extra stimuli), Figure 3. In the case of inducible VTs, a new mapping and ablation was performed until non-inducibility was obtained.

Extracorporeal membrane oxygenation (ECMO) support was increased during acute hemodynamic decompensation due to spontaneous or induced VT to permit optimal mapping and ablation of arrhythmias. Procedural success was defined as an inability to induce sustained VTs and the disappearance of frequent spontaneous premature ventricular complexes.

In all the patients, the arterial and venous cannulas were removed at the end of the procedure. The right femoral artery was closed using the previously positioned Perclose Proglide closure system and the vein was closed with manual compression. In four patients, ECMO cannulas were removed by vascular surgeons. After ECMO cannula removal, right femoral artery angiography was performed to exclude the possibility of procedural damage (the angiography catheter was inserted through the left femoral artery). Blood inside of the circuit was recovered using an autologous blood recovery machine (Cell Saver^®^5+, Haemonetics Corporation, Boston, Massachusetts, United States) and infused into the patient.

### 2.3. Follow-Up

Clinical follow-up was performed every month after catheter ablation for the first year, then every 6 months by clinical evaluation, echocardiogram and ICD checkup. Clinical recurrence, ICD therapy and procedural complications were recorded for all patients.

### 2.4. Endpoints

The primary endpoint is overall cardiovascular death, including death due to heart failure, cardiogenic shock or ventricular arrhythmias. In particular, arrhythmic death was defined as death occurring during an ES.

Secondary endpoints include arrhythmic recurrences, hospitalizations, left ventricular assist device implantation, and heart transplantation. Arrhythmic recurrences were defined as sustained VT/VF with an ICD intervention (ATP or shock).

Primary endpoints were compared between the ischemic cardiomyopathy group and non-ischemic cardiomyopathy patients.

### 2.5. Statistical Analysis

Continuous variables were expressed as mean (M) and standard deviation (SD) or median and interquartile range, while categorical variables were expressed as number (n) and percentage (%). A Kaplan–Meier curve was performed to evaluate primary endpoints and arrhythmic recurrences. The group’s outcome was assessed by using the log-rank test. A *p*-value < 0.05 was considered statistically significant. Statistical analysis was performed with SPSS Statistics version 22 (IBM Corporation, City, State if Canada/USA, Country).

## 3. Results

### 3.1. Study Population

The population of this retrospective study included 47 patients, including 42 males (89.4%), mean age 66.7 ± 9.6 years. Baseline characteristics of the study population are reported in Table 1. Twenty-five patients (53.2%) were affected by ischemic cardiomyopathy; the remaining suffered from dilated cardiomyopathy (36.2%), idiopathic ventricular fibrillation (4.3%), myocarditis (2.1%), arrhythmogenic right ventricular cardiomyopathy (2.1%) and hypertrophic cardiomyopathy (2.1%). Heart failure was documented in 44 cases (93.6%); mean left ventricular ejection fraction was 31.5 ± 9.9% and mean left ventricular end diastolic diameter was 62.2 ± 7.6 mm.

Forty-two patients (89.4%) were admitted or referred due to electrical storm and median PAINESD score [13] was 17 (13–23).

Considering risk factors, 11 patients (23.4%) had a family history of sudden cardiac death, hypertension was present in 21 (44.7%), diabetes in nine (9.1%), dyslipidemia in 26 (55.3%) and chronic kidney disease in nine (9.1%). Moreover, 14 people (29.8%) were smokers and five (10.6%) were affected by chronic obstructive pulmonary disease.

Among patients with ischemic heart disease, 23 (48.9%) underwent percutaneous coronary intervention and three (6.4%) coronary artery bypass graft.

At hospital admission, beta blockers were taken by 43 patients (91.5%) and antiarrhythmic drugs by 38 (80.9%); concerning heart failure therapy, 23 subjects (48.9%) took ACE (angiotensin converting enzyme) inhibitors or angiotensin receptor blockers, 15 (32.6%) angiotensin receptor/neprilysin inhibitors (ARNI) and seven (14.9%) sodium-glucose transporter 2 inhibitors (SGLT2). Diuretics were prescribed in 38 cases (80.9%). Twenty-four patients (51.1%) took antiplatelets and 24 (51.1%) anticoagulants.

In 45 patients, implantable cardioverter defibrillator (ICD) had been previously implanted; 74.5% were single or dual chamber ICD and 21.3% biventricular ICD for cardiac resynchronization therapy.

### 3.2. Procedural Data

Procedural data are reported in Table 2. In the overall population, mean clinical tachycardia cycle length was 296 ± 99 ms; three patients (6.4%) were scheduled because of ventricular fibrillation (VF), comprising two cases of idiopathic VF and one case of post-infarction Purkinje-triggered VF. In 11 cases (23.4%) epicardial access and ablation were needed.

Mean procedural time was 255 ± 108 min with 13.2 ± 9.1 min fluoroscopy time and 50 (22–86) G/cm^2^ dose area product. Acute success was achieved in 43 patients (91.4%), considering 39 cases of no arrhythmia inducibility (82.9%), four cases of non-sustained ventricular tachycardia induction and four of VT/VF induction. Two patients underwent early re-do in the same hospitalization.

After the indexed procedure, complications occurred in four cases (8.5%) including two cardiac tamponades, one hemorrhagic shock and one femoral stenosis. Cases of cardiac tamponade were treated with pericardial drainage and femoral stenosis was cured with percutaneous transluminal angioplasty (Table 3). None of these patients have died due to procedural complication.

Considering the hospital stay, infective status occurred in seven patients (14.9%), among whom four died; hospital stay lasted 11.7 ± 6 days mean and 87.2% of patients were discharged to home, while 10.6% were transferred to rehabilitation. One patient died in the hospital.

### 3.3. Outcomes

**Figure 4:**
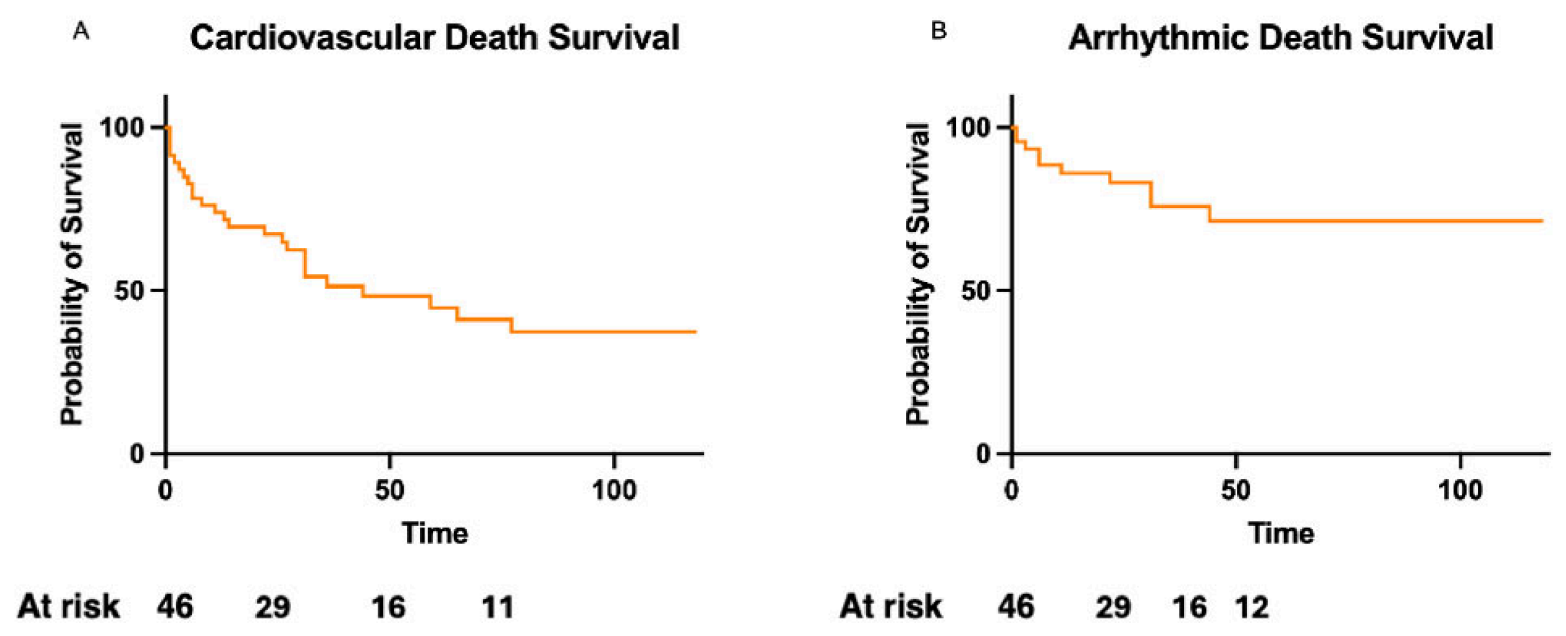
Kaplan–Meier curve of cardiovascular death (**A**) and arrhythmic death (**B**).

After a median follow-up of 28 (7–63.5) months, cardiovascular death occurred in 26 patients (55.3%), as shown in Table 4 and Figure 4A, but arrhythmic death befell only 11 patients (23.4%); see Figure 4B. All deaths occurred within 6–7 years of follow-up. All follow-up data are reported in Figure 4 and Table 4. No patient was assessed by cardiac surgeons as suitable for LVAD implantation.

**Figure 5:**
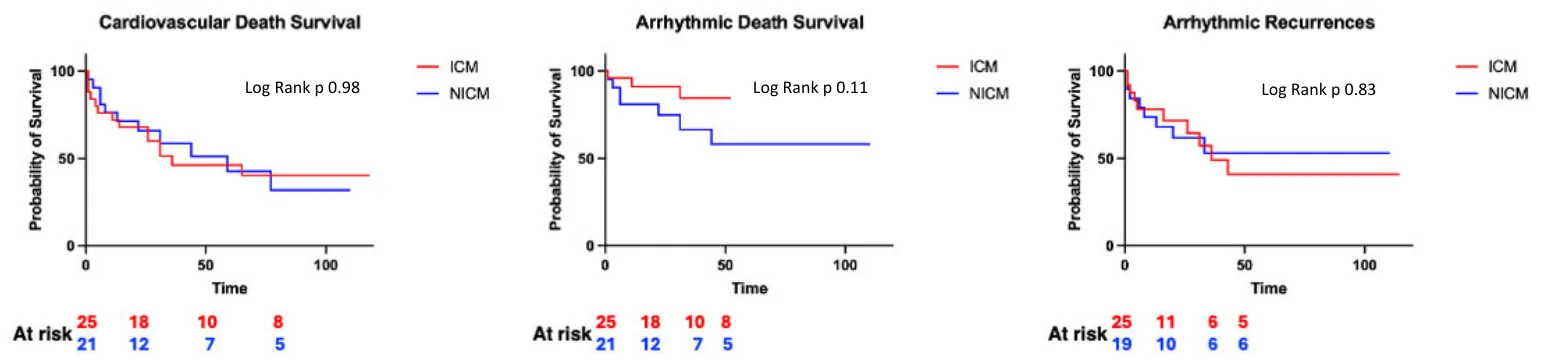
Kaplan–Meier curve comparison of cardiovascular death, arrhythmic death and arrhythmic recurrences between ischemic cardiomyopathy and non-ischemic cardiomyopathy.

Of note, no difference exists between ischemic and non-ischemic cardiomyopathy with regard to primary endpoints (Figure 5).

**Figure 6:**
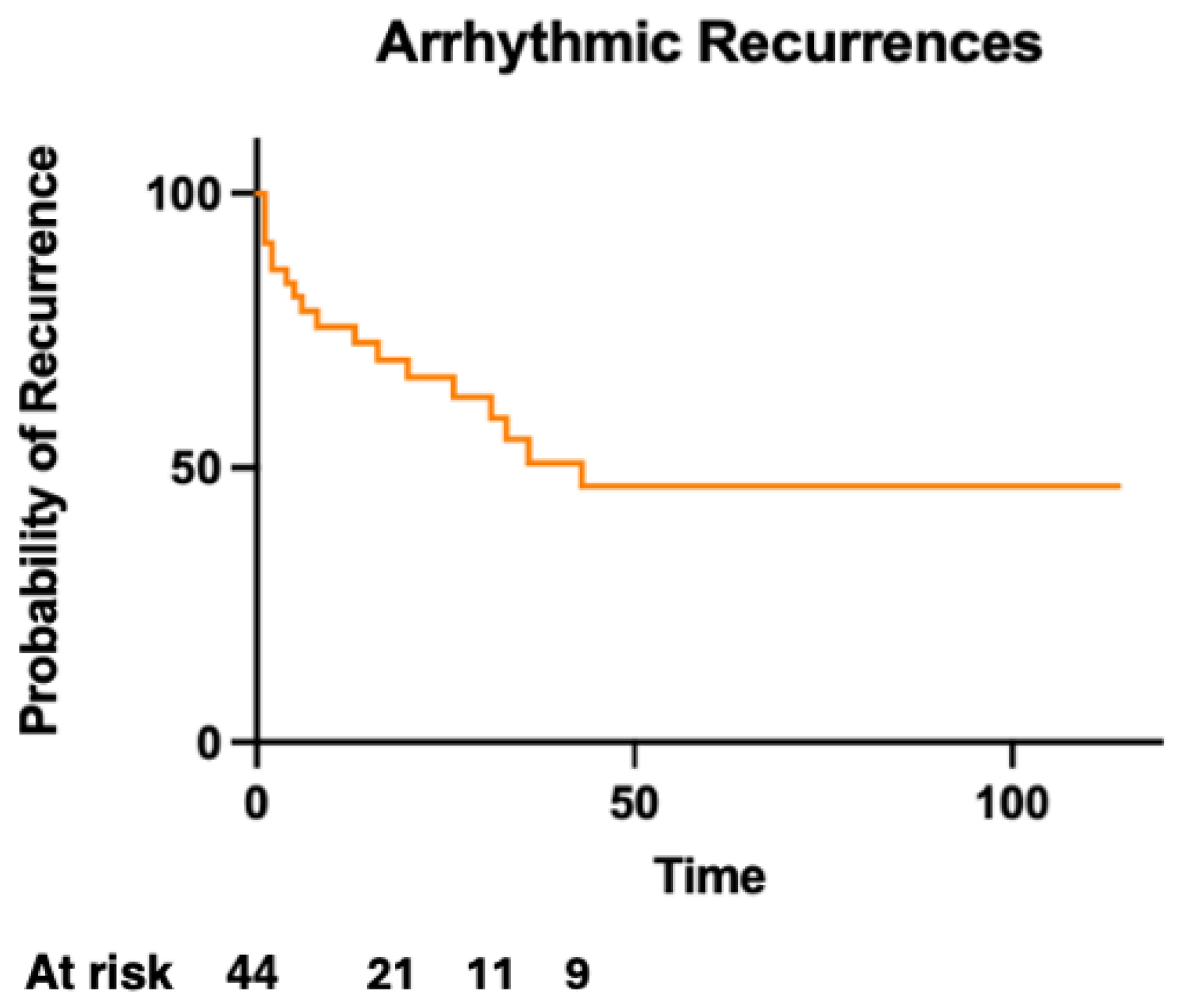
Kaplan–Meier curve of arrhythmic recurrences.

Moreover, arrhythmic recurrences occurred in 21 patients (44.7%), among whom only 15 (31.9%) had ICD shocks (Figure 6).

Twenty-five patients (55.3%) encountered further hospitalizations: in seven cases a re-do procedure was performed, and in three patients CIEDs (cardiovascular implantable electronic device) upgrading was chosen. Regarding final pathways of advanced heart failure, no patients were referred for left ventricular assist device, three patients (6.4%) were put on the transplant list and six (12.8%) underwent cardiac transplant.

## 4. Discussion

This retrospective study investigated the long-term follow-up of ventricular arrhythmias catheter ablation supported by extracorporeal membrane oxygenation. The findings of this work demonstrate that:

- ECMO support is useful to prevent hemodynamic instability and procedural heart failure during ablation of unstable VAs.
- Catheter ablations supported by ECMO show acceptable arrhythmic outcomes despite substantial competing mortality from advanced heart failure.
- Early ECMO weaning and removal guarantees low rate of complications, avoiding longer hospital stay.

Catheter ablation has become an established treatment of ventricular arrhythmias, especially in patients with structural heart disease and mainly with ischemic heart disease [1,10,29]. Ablation decreases arrhythmia burden and ICD shocks, improves the quality of life, and reduces the need for antiarrhythmic drugs [3,6,8,9,10,11,12]. Moreover, it could be vital for patients with unstable or refractory VAs or electrical storm [3,6,7,30,31]. But we already mentioned that these procedures could be complicated by hemodynamic deterioration and acute heart failure occurrence since patients are often fragile with advanced heart failure [13]. Indeed, hemodynamic instability could be led by several factors such as the need for unstable arrhythmia inducibility and mapping during the procedure, fluid overload, multiple electrical cardioversions, catheter movements and pacing maneuvers.

This problem could be managed with ECMO support as demonstrated by several studies in the literature, from the very early experiences [15,32] using intermittent mechanical support to the most recent evidence [16,17,19,20,21,22,33]. Numerous strategies are available, including pre-emptive use of ECMO [13,16,19,20,34], rescue mechanical support [18,33] and bail-out strategy [17]. The last two approaches failed to demonstrate real advantages, especially in the case of the rescue one, whereas pre-emptive use of cardiopulmonary support showed promising results. We previously published our experience of catheter ablations supported by pre-emptive ECMO in patients with unstable ventricular tachycardias or electrical storm, proving acute heart failure prevention and safety [16].

However, ECMO is not only important for hemodynamics, since it was demonstrated to be effective in allowing successful mapping and ablation of arrhythmias. This outcome could be explained by the ability to perform more extensive inducibility targeting multiple arrhythmias and more accurate VAs mapping and entrainment thanks to the patient stability granted by ECMO [20,21].

Some groups try to avoid hemodynamic instability upstream, pointing out the higher risk of complications of cardiopulmonary support and of general anesthesia and suggesting a more conservative approach based on substrate mapping and modification [35,36,37]. Additionally, they advocate for the referral of more critical and advanced patients, perhaps with unstable VAs and electrical storm, to left ventricular assist devices and transplant without performing any attempts of catheter ablation.

In this study, we propose a different approach, based on long experience and on clinical outcomes from literature evidence. The first step is the selection of the patient, who cannot be too severe or fragile, otherwise they should be guided to palliative care or mechanical support without ablation as electrical storm management and a bridge to candidacy or a transplant. From this perspective, inclusion criteria for ECMO-supported ablation would be fast VT or VF, ventricular arrhythmia associated with hemodynamic impairment, several VT morphologies documented, and PAINESD > 17. Patients older than 85 years old, too fragile according to available scores with unsuitable vascular access, multiorgan failure, severe chronic kidney injury, especially in dialysis, polymorphic VT, EF < 20%, arrhythmias derived from mechanical cardiogenic shock or acute heart failure with indication for LVAD/heart transplant, must be ruled out from this kind of procedure. This choice avoids very high-risk procedures or difficulties in ECMO weaning. Obviously, we also need to identify the right candidate for ECMO support, balancing benefits and risks; some scores and studies help us with this decision.

The second step is the avoidance of general anesthesia and its possible complications, preferring deep sedation with comprehensive monitoring. Finally, we look for non-inducibility of ventricular arrhythmias as a main goal of the procedure since there are several studies that demonstrate good outcomes and prognosis for these patients [21,38,39,40].

To the best of our knowledge, this is the first study to present long-term results of this approach.

Baratto et al. [20] have previously demonstrated the safety and efficacy of ECMO support for catheter ablation, including 64 patients and 74 procedures. They obtained acute success in 79–81% of patients with a median procedural time of 300 min (210–360) and 1.5% early mortality, while the 12-month follow-up showed 77% freedom from arrhythmic recurrences with lower rate in the non-inducible group and 12% overall mortality. On the other hand, a multicenter American collaborative group study [22], including 1655 patients, compared patients who received hemodynamic support during procedure (105, Impella, TandemHeart and ECMO) with those who did not. They reported that patients with hemodynamic support were sicker, with multiple comorbidities, and had higher in-hospital and long-term mortality rate after more than one year of follow-up (21% and 34.7% respectively). In this case long-term procedural success was achieved in 71.8% with 296 min of median procedural time, at the price of more complications when compared to the control group. Similar results were presented by an Australian group [19]. Other groups tried to use different mechanical circulatory support using Impella, TandemHeart^®^ or LVAD, but results are not homogenous, showing some drawbacks, especially with Impella^®^ [18,34,38,41,42,43,44,45].

In this study, no periprocedural heart failure or hemodynamic impairment event occurred. More importantly, we obtained 91.4% acute success and only 8.5% still-inducible non-sustained arrhythmias. Considering follow-up, freedom from VAs recurrences was 70.9% at one year, similar to previous data; it worsened during following months, reaching a 55.3% long-term success rate at almost three years of median follow-up. Interestingly, ICD shocks were relatively lower, meaning that less unstable or fast VAs occurred, making non-supported procedures and substrate modification more feasible.

Most of the patients included in this cohort could be classified as advanced or worsening heart failure patients, explaining the higher overall cardiovascular mortality rate, considering still-good results at one year with more than 70% survival. Moreover, our population is slightly older and has a poorer mechanical heart function as compared to those from other studies.

Nevertheless, one of the most interesting results is arrhythmic death that is significantly lower, demonstrating that patients died more due to mechanical dysfunction associated with the worsening of the underlying disease than because of a fatal arrhythmic recurrence.

Beyond clinical outcomes, organization and workflow seem to be crucial. Indeed, the use of pre-emptive ECMO and early weaning and removal guarantee less complications. This result could also be explained by shorter procedural time.

Some limitations must be addressed. Indeed, this is a single-center retrospective study with no randomized design. Furthermore, the number of patients is relatively small without a control group and procedures were performed by experienced operators using different types of mapping and ablation tools, influenced by technological development over time.

## 5. Conclusions

ECMO may facilitate unstable or fast ventricular arrhythmia mapping and ablation, showing good acute procedural success. However, the impact on the efficacy and safety of ECMO support during catheter ablation requires further studies.

## Figures and Tables

**Table 1:** Baseline characteristics of the study population.

**Variables**	**Overall Population (*n* = 47)**
Age, y	66.7 ± 9.6
Male, *n* (%)	42 (89.4)
BMI, kg/m^2^	23.6 ± 5.5
PAINESD Score	17 (13–23)
Electrical Storm, *n* (%)	42 (89.4)
Ischemic Cardiomyopathy, *n* (%)	25 (53.2)
Hypertrophic Cardiomyopathy, *n* (%)	1 (2.1)
Dilated Cardiomyopathy, *n* (%)	17 (36.2)
ARVC, *n* (%)	1 (2.1)
Myocarditis, *n* (%)	1 (2.1)
Idiopathic VF, *n* (%)	2 (4.3)
Heart Failure, *n* (%)	44 (93.6)
Family History of SCD, *n* (%)	11 (23.4)
Hypertension, *n* (%)	21 (44.7)
Diabetes, *n* (%)	9 (19.1)
Smokers, *n* (%)	14 (29.8)
Dyslipidaemia, *n* (%)	26 (55.3)
CKD, *n* (%)	9 (19.1)
COPD, *n* (%)	5 (10.6)
PCI, *n* (%)	23 (48.9)
CABG, *n* (%)	3 (6.4)
Heart Valve Surgery, *n* (%)	1 (2.1)
Thyroid Dysfunction, *n* (%)	8 (17)
Stroke, *n* (%)	3 (6.4)
Chronic Inflammatory Disease, *n* (%)	5 (10.6)
Drugs at Hospital Admission	
Beta Blockers, *n* (%)	43 (91.5)
ACE Inhibitors/ARBS, *n* (%)	23 (48.9)
ARNI, *n* (%)	15 (32.6)
SGLT2 Inhibitors, *n* (%)	7 (14.9)
Diuretics, *n* (%)	38 (80.9)
Antiarrhythmic Drugs, *n* (%)	38 (80.9)
Antiplatelets, *n* (%)	24 (51.1)
Anticoagulants, *n* (%)	24 (51.1)
LVEF, %	31.5 ± 9.9
LVEDD, mm	62.2 ± 7.6
CIEDs	-
Single/Dual Chamber ICD, *n* (%)	35 (74.5)
Biventricular ICD, *n* (%)	10 (21.3)
TCL, ms	296 ± 99
VF, *n* (%)	3 (6.4)

Data are presented as: mean ± standard deviation, median (quartile) or n (percentage). BMI: body mass index; PAINESD score: age > 60 anni, NYHA III/IV, electrical storm, diabetes, ejection fraction < 20%, COPD; ARVC: arrhythmogenic right ventricular cardiomyopathy; VF: ventricular fibrillation; SCD: sudden cardiac death; CKD: chronic kidney disease; COPD: chronic obstructive pulmonary disease; PCI: percutaneous coronary intervention; CABG: coronary artery bypass graft; SGLT2 inhibitors: sodium/glucose transporter 2 inhibitors; ACE: angiotensin converting enzyme; ARBs: angiotensin receptor blockers; ARNI: angiotensin receptor/neprilysin inhibitors; LVEF: left ventricular ejection fraction; LVEDD: left ventricular end diastolic diameteres; CIEDs: cardiac implantable electronic devices; ICD: implantable cardioverter defibrillator; and TCL: tachycardia cycle lenght.

**Table 2:** Procedural data.

Variables	Overall Population (*n* = 47)
Procedural Time, min	255 ± 108
Fluoroscopy Time, min	13.2 ± 9.1
Dose Area Product, G/cm^2^	50 (22–86)
Epicardial Ablation, *n* (%)	11 (23.4)
Post Procedural Inducibility	
None, *n* (%)	39 (82.9)
NSVT, *n* (%)	4 (8.5)
VT/VF, *n* (%)	4 (8.5)
Acute Success, *N* (%)	43 (91.4)
Early Re-Do *, *n* (%)	2 (4.3)
Hospital Stay, d	11 (7.5–14)
Discharge	
Home, *n* (%)	41 (87.2)
Rehabilitation, *n* (%)	5 (10.6)

Data are presented as: mean ± standard deviation, median (quartile) or n (percentage). * Re-do during the same hospitalization of the indexed procedure.

**Table 3:** Complications.

Variables	Overall Population (*n* = 47)
Bleeding, *n* (%)	1 (2.1)
Cardiac Tamponade, *n* (%)	2 (4.3)
Vascular Complications, *n* (%)	1 (2.1)
Limb Ischemia, *n* (%)	0 (0)
Blood Transfusion, *n* (%)	1 (2.1)
Infections, *n* (%)	7 (14.9)
Renal Failure, *n* (%)	0 (0)
Stroke, *n* (%)	0 (0)
Prolonged ECMO, *N* (%)	0 (0)
In-hospital Mortality, *n* (%)	1 (2.1)

Data are presented as: mean ± standard deviation, median (quartile) or n (percentage). ECMO: Extracorporeal membrane oxygenation.

**Table 4:** Follow-up data.

Variables	Overall Population (*n* = 47)
Follow-up, m	28 (7–63.5)
Arrhythmic Recurrences, *n* (%)	21 (44.7)
ICD Shocks, *n* (%)	15 (31.9)
LVAD, *n* (%)	-
Transplant List, *n* (%)	3 (6.4)
Heart Transplant, *n* (%)	6 (12.8)
New Hospitalization, *n* (%)	25 (55.3)
Late Re-Do *, *n* (%)	7 (14.9)
CIEDs Upgrading, (%)	3 (6.4)
Arrhythmic Death, *n* (%)	11 (23.4)
Overall Cardiovascular Death, *n* (%)	26 (55.3)

Data are presented as: mean ± standard deviation, median (quartile) or n (percentage). ICD: implantable cardioverter defibrillator; LVAD: left ventricular assist device; CIEDs: cardiac implantable electronic devices. * Re-do after hospital discharge.

## Data Availability

The data presented in this study are available on request from the corresponding author due to privacy and ethical reasons.

## Abbreviations

The following abbreviations are used in this manuscript:

ACE	Angiotensin Converting Enzyme
ARNI	Angiotensin Receptor Neprilysin Inhibitor
CI	Confidence Interval
CIEDs	Cardiovascular Implantable Electronic Devices
ECMO	Extracorporeal Membrane Oxygenation
ES	Electrical Storm
ICD	Implantable Cardioverter Defibrillator
LVAD	Left Ventricular Assist Device
SGLT2	Sodium Glucose Transporter 2
VAs	Ventricular Arrhythmias
VF	Ventricular Fibrillation
VT	Ventricular Tachycardia
